# Real-world heart failure management in 10,910 patients with chronic heart failure in the Netherlands: Design and rationale of the *C*hronic *H*eart failure *E*SC guideline-based *C*ardiology practice Quality project (CHECK-HF) registry

**DOI:** 10.1007/s12471-018-1103-7

**Published:** 2018-03-21

**Authors:** J. J. Brugts, G. C. M. Linssen, A. W. Hoes, H. P. Brunner-La Rocca, CHECK-HF investigators, H. van >Amerongen, A. Derks, D. Hering, H. J. Kruik, M. Martherus, J. Pluimers, C. E. M. Rodijk-Heijmer, A. Uitzetter, D. Veldhuis, N. A. M. Huisman, A. van der Spank, J. Winter, A. H. M. Moons, M. Smit, R. M. Oortman, N. Aengenend, H. J. J. Koornstra-Wortel, T. Rongen, K. J. Balhuizen, J. Plomp, A. A. M. van Drimmelen, I. Snoek, A. van Anken, L. van Rijn, F. J. J. Smeele, M. W. F. van Gent, G. C. van Lingen-Koppejan, P. A. Smits, H. I. S. Trossèl, H. J. Schaafsma, G. Tuin-v.d. Kolk, H.D. Vermeulen-v.d. Wetering, J. Zimmerman, A. Adema, J. W. Brakel, M. J. Nagelsmit, W. Veenstra, I. Aksoy, D. C. Meulmeester-Sinke, P. S. Monraats, H. H. Reijnierse-Buitenwerf, A. H. Witkam-Bal, M. Boes-van Laar, H. M. C. Schoep-Bezemer, P. H. M. Westendorp, A. van Dieën, E. P. Viergever, E. B. Vossebelt, L. H. Takens, W. E. H. de Valk-Bedijn, C. L. B. van der Bolt, R. Hendrick, J. A. Kragten, N. P. Stoot, M. A. Barandiaran Aizpurua, N. G. H. M. Marcks, J. Merken, L. Corsten, J. C. Kelder, R. M. van Tooren, T. Hillebrink, L. Oosterom, N. Telgt, B. M. van Dalen, A. van Miltenburg, N. Slingerland, B. Sonneveld, E. Bird-Lake, J. Hoek-Verschoor, A. van der Ree, A. Erol-Yilmaz, L. den Hartog-Taai, P. Middelburg-Poldervaart, P. C. Rademaker, S. de Smet, E. G. M. V. de Theije, T. J. de Wit, J. Langerveld, C. J. Morang-van Drempt, M. M. Vermeulen, Y. Foolen, A. C. B. Pronk, B. M. Szabó, L. K. Valk, M. J. W. Grosfeld, M. Aertsen, D. J. M. Engelen, V. Kneijber, J. van Santvoord, L. W. M. Eurlings, E. J. Geurts, R. Hazeleger, A. M. Koopman-Verhagen, G. Maessen, C. W. A. M. Pansters, P. R. Geerlings, M. de Boer, A. Kolkman, C. van der Lee, R. Blonk, J. Krijger, J. P. P. Smits, N. Y. Y. Al-Windy, M. Harmsen

**Affiliations:** 1 Department of Cardiology, Erasmus Medical Center, Thoraxcenter, Rotterdam, The Netherlands; 2 Department of Cardiology, Hospital Group Twente, Almelo and Hengelo, The Netherlands; 3 Julius Center for Health Sciences and Primary Care, Utrecht, The Netherlands; 4 University Medical Center Utrecht and Utrecht University, Utrecht, The Netherlands; 5 Department of Cardiology, Maastricht University Medical Center, Maastricht, The Netherlands

**Keywords:** Heart failure, Guideline, Adherence, Registry

## Abstract

**Aims:**

Data from patient registries give insight into the management of patients with heart failure (HF), but actual data from unselected real-world HF patients are scarce. Therefore, we performed a cross sectional study of current HF care in the period 2013–2016 among more than 10,000 unselected HF patients at HF outpatient clinics in the Netherlands.

**Methods:**

In 34 participating centres, all 10,910 patients with chronic HF treated at cardiology centres were included in the CHECK-HF registry. Of these, most (96%) were managed at a specific HF outpatient clinic. Heart failure was typically diagnosed according to the ESC guidelines 2012, based on signs, symptoms and structural and/or functional cardiac abnormalities. Information on diagnostics, treatment and co-morbidities were recorded, with specific focus on drug therapy and devices. In our cohort, the mean age was 73 years (SD 12) and 60% were male. Frequent co-morbidities reported in the patient records were diabetes mellitus 30%, hypertension 43%, COPD 19%, and renal insufficiency 58%. In 47% of the patients, ischaemia was the origin of HF. In our registry, the prevalence of HF with preserved ejection fraction was 21%.

**Conclusion:**

The CHECK-HF registry will provide insight into the current, real world management of patient with chronic HF, including HF with reduced ejection fraction, preserved ejection fraction and mid-range ejection fraction, that will help define ways to improve quality of care. Drug and device therapy and guideline adherence as well as interactions with age, gender and co-morbidities will receive specific attention.

## Introduction

Chronic heart failure (HF) is an important health care problem with high cardiovascular morbidity and mortality [1]. The prevalence of HF is 1–2% in developed countries and is expected to rise even further in the next decades [2]. Chronic HF has a disturbing prognosis if untreated or insufficiently treated, while suboptimal treatment is often not sufficiently recognised [3, 4].

We could improve the prognosis considerably when we would optimise drug and device therapy according to the European Society of Cardiology (ESC) guidelines [5], at least in the patients with HF and reduced left ventricular ejection fraction (HFrEF). Optimal treatment also includes adequate attention to lifestyle habits and management of co-morbidities. Considering medication, a guideline-based approach directly enhances the quality of care delivered to HF patients. However, older surveys such as the EuroHeart survey showed that only a minority of the patients were treated adequately with these agents, and when considering target dose the figures are even worse [6]. More recent surveys show much better adherence to HF treatment, but these surveys have shortcomings such as limited representativeness of included patients or lack of information on drug dose [7–9]. Furthermore, most studies did not distinguish between HFrEF and HF with preserved LVEF (HFpEF) [7–9]. We should consider these aspects if we want to assess adherence to guidelines in daily practice, which is a clear marker of quality of care delivered by health care professionals. Suboptimal adherence to guidelines is often claimed to be due to the presence of co-morbidities, but this has not been sufficiently studied. Therefore, it is important that we enhance awareness of the complexity of modern HF care, which includes the management of co-morbidities.

For better insight into these clinically relevant aspects, we set up a large cross-sectional study to assess current treatment of HF patients at outpatient clinics. The aim of the study was to describe clinical characteristics and treatment of unselected patients with HF in the Netherlands. Notably, we will evaluate how recommendations of the European guidelines regarding pharmacological and non-pharmacological treatments are adopted in clinical practice and to find determinants of non-adherence.

### The CHECK-HF study design

The CHECK-HF (*Chronisch Hartfalen ESC-richtlijn Cardiologische praktijk Kwaliteitsproject-HartFalen*) is a Dutch registry aimed at improving quality of HF care in the Netherlands.

**Fig. 1:**
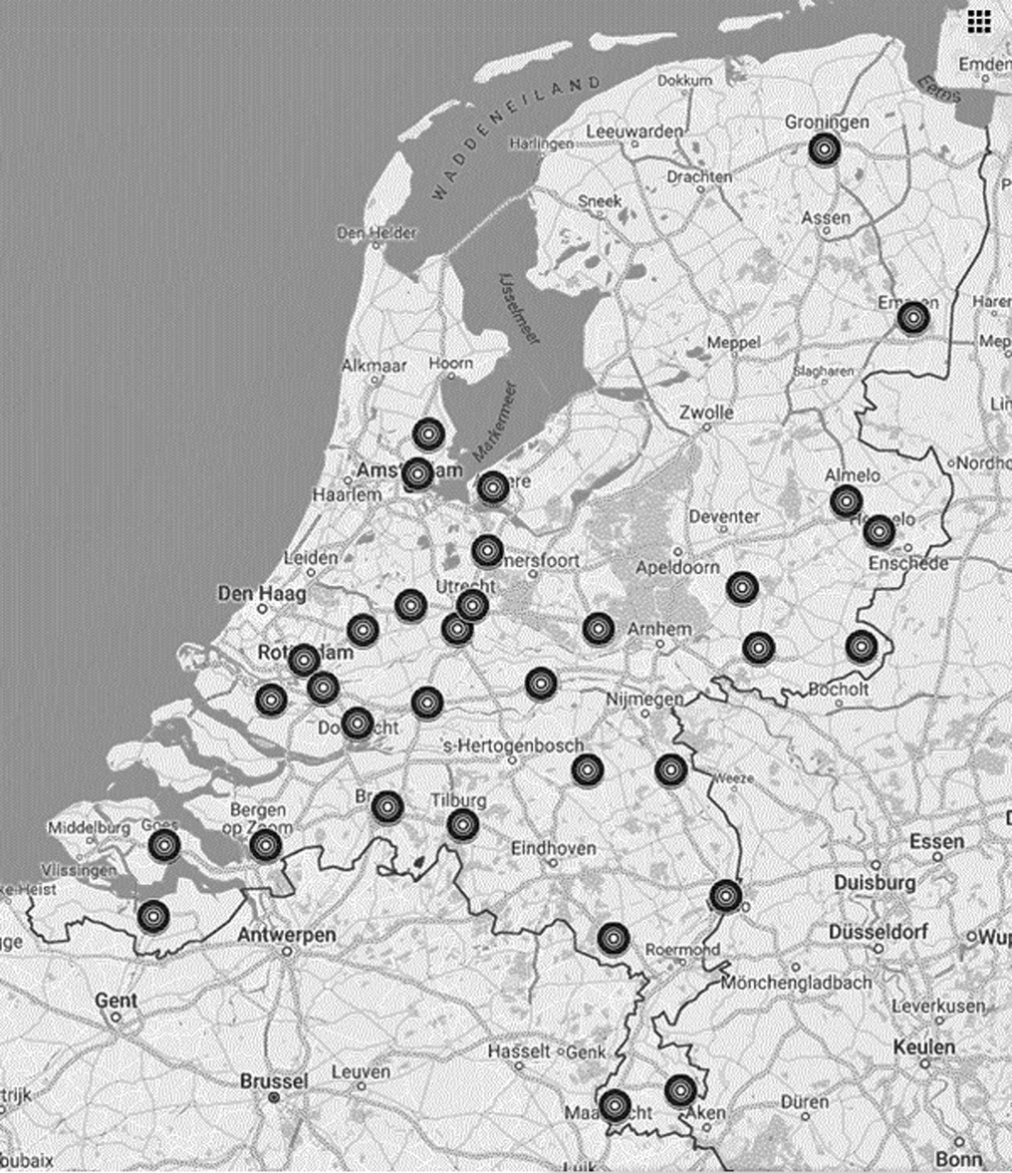
The 34 participating centres in the Netherlands

The current study is a cross-sectional registration of all unselected patients diagnosed with chronic HF who are treated at Dutch outpatient HF clinics (96%) in the period September 2013–September 2016. All patients with HF seen at the general cardiology outpatient clinic of the same hospitals (4%) could be included as well. There are no available data that allow us to estimate the exact proportion of patients from the centres that was included in our database. The objective was to include all enlisted patients. In view of the estimated average number of patients per centre the average proportion of included outpatient clinic patients with HF is well above 80%. In total, 34 Dutch hospitals, including 1 academic hospital agreed to participate (Supplementary material list of participating centres and investigators) (Fig. 1).

The ethics committee approved our study (METC 2017 – Maastricht University Medical Center, Maastricht, the Netherlands).

## Type of patients

Patient were included if the diagnosis of HF was based on the criteria of the ESC guidelines, that is signs and symptoms, and structural and/or functional cardiac abnormalities [5]. Heart failure was subtyped in HFrEF (EF < 50%) or HFpEF (EF ≥ 50%) according to the guidelines that were in force at that time. Baseline ejection fraction assessments by echocardiography were available for the majority of patients at inclusion, while in some this was only classified semi-quantitatively (in a very small proportion, information was not provided). In the patients with available % ejection fraction, the whole spectrum of LVEF according to the new guidelines classification including the new group of mid-range EF (HFmrEF) with an EF of 40 to 49% can be studied [10]. No further exclusion criteria were applied apart from age <18 years. Aetiology of HF was further defined as ischaemic or non-ischaemic and further specified as hypertensive, valvular, related to arrhythmias or other, if applicable.

## Baseline characteristics and demographics

The recorded dataset at inclusion consisted of age, sex, length, weight, body mass index, heart rate, systolic blood pressure, diastolic blood pressure, date of diagnosis, date of first outpatient visit, date of inclusion, New York Heart Association (NYHA) class, EF, HFrEF or HFpEF, pacemaker, implantable cardioverter defibrillator (ICD) or cardiac resynchronisation therapy (CRT), QRS duration, basic rhythm, laboratory values (serum creatinine, urea, potassium, sodium, brain natriuretic peptide (BNP), N‑terminal pro hormone BNP (NT-proBNP)), co-morbidity and participation in cardiac rehabilitation programme.

## Assessment of current medication use

The recorded dataset includes in-depth information (type, dosage and frequency, total daily dose) of the use of beta-blockers, angiotensin converting enzyme inhibitors (ACE) or angiotensin receptor II antagonists (ARB), mineralocorticoid receptor antagonists (MRA), ivabradine, diuretics (furosemide, bumetanide, hydrochlorothiazide, triamterene), digoxin, amiodarone, and statins. The use of platelet inhibitors and/or oral anticoagulants (yes/no) was recorded as well.

## Echocardiographic parameters

At inclusion, we recorded values of the most recent echocardiogram of the patient with estimated ejection fraction in percentage either by biplane Simpson or Teicholtz, or left ventricular function semi-quantitatively assessed as normal function or mildly, moderately or severely impaired function. In the majority of patients, quantitative values of EF were available and recorded (73%). Diastolic function, presence of restrictive filling pattern of the left ventricle as well as semi-quantitative assessment of inferior vena cava dimension/collapse, and pressure gradient across the tricuspid values were recorded.

## Co-morbidities

The recorded dataset specifically noted the presence of co-morbidities as recorded in medical history (diabetes mellitus, hypertension, hypercholesterolaemia, renal insufficiency (estimated glomerular filtration rate <60), anaemia (haemoglobin below age-dependent threshold), thyroid dysfunction, chronic obstructive pulmonary disease (COPD) or obstructive sleep apnoea, and peripheral arterial occlusive disease).

## Data inclusion

Data inclusion was performed using the CHECK-HF database based on Microsoft Excel with Visual Basic for Application designed to digitalise characteristics of patients and patient flow at the outpatient HF clinics. Each hospital recorded their data in a uniform stand-alone online database. Patient data were encoded and exported to a central database, fully anonymised, and cannot be traced to an individual patient. Data inclusion was based on a standardised protocol including a set of variables. As data collection was based on daily practice not all variables were available in all patients.

## Outcome measures

The main analysis will present the adherence to guideline-directed medication-based therapy and device-based therapy in HFrEF patients. We will analyse adherence as percentage (%) of prescriptions as well as percentage (%) of target dose of guideline-recommended heart failure treatments [5]. In addition, we will analyse the percentage of prescribed ICD therapy and CRT defibrillator (CRT-D) therapy. Adherence data will be presented in future manuscripts. Secondary analyses will focus on specific subgroups of reduced and preserved left ventricular ejection fraction (LVEF) as well as comorbidities.

## Statistical analysis

Summary statistics are provided as percentages (%) or as mean with standard deviations (SD).

Baseline continuous variables are presented as mean ± SD or median and interquartile range, depending on the distribution of the data; categorical data are presented as counts and percentages. We will compare the categorical variables using the χ^2^ test and the continuous variables using the *t*-test or the Mann-Whitney *U*-test, as appropriate. Univariate and multivariate regression analysis will be used to calculate odds ratio and 95% confidence interval. Drug dose will be calculated compared with the recommended dose according to guidelines as percentage of actual recommended daily dose. Analyses are and will be performed with SPSS system software (version 24.0 or later).

## Results

Demographics of the enrolled patients can be found in Tab. 1. About 60% were male and the average age was 73 years, with a wide range between 18 and 103 years of age (median 75 years, interquartile range (IQR) 66–82 years). Co-morbidities were common and in about half of the patients their HF was of ischaemic origin (46%). The vast majority of patients had reduced left ventricular ejection fraction (79%). Mean ejection fraction was 37% (SD 14). Most patients were in functional NYHA class II (55%).

**Table 1:** Baseline demographics in 10,910 patients with chronic heart failure (CHECK-HF)

Baseline characteristics	
Age, years (mean, SD); *n* = 10,890	72.8 ± 11.9
Gender, % male; *n* = 10,859	6523 (60.1%)
Body mass index, kg/m^2^ (mean, SD); *n* = 9989	27.5 ± 5.4
Hypertension, %; *n* = 9733	4173 (42.9%)
Diabetes mellitus, %; *n* = 9733	2877 (29.6%)
Renal insufficiency^a^, %; *n* = 8690	5055 (58.2%)
COPD, %; *n* = 9733	1829 (18.8%)
*Aetiology: n* *=* *10,468*	
Ischaemic cardiomyopathy, %	4877 (46.6%)
Non-ischaemic cardiomyopathy, %	5591 (53.4%)
*Heart failure: n* *=* *10,627*	
Reduced ejection fraction, %	8360 (78.7%)
Preserved ejection fraction, %	2267 (21.3%)
Unknown, %	283 (2.6%)
LVEF, % (mean, SD); *n* = 9178	36.0 ± 14.1
*NYHA class; n* *=* *10,664*	
I, %	1808 (17.0%)
II, %	5884 (55.2%)
III, %	2777 (26.0%)
IV, %	195 (1.8%)
Heart rate, bpm (mean, SD); *n* = 10,659	72.2 ± 14.1
Systolic BP, mm Hg (mean, SD); *n* = 10,664	127.6 ± 21.5
Heart failure outpatient clinic; *n* = 10,780	10,385 (96.3%)

*BP* blood pressure, *bpm* beats per minute, *COPD* chronic obstructive pulmonary disease, *eGFR* estimated glomerular filtration rate*, HF* heart failure, *LVEF* left ventricular ejection fraction, *MI* myocardial infarction, *NYHA* New York Heart Association, *SD* standard deviation

^a^Defined as eGFR below 60 or history of renal failure

## Discussion

The current study will evaluate current quality of HF care in unselected patients with heart failure in the Netherlands. The CHECK-HF registry will demonstrate current adherence to ESC guidelines in the period of 2013–2016, that is between the publications of the 2012 and 2016 guidelines [5, 10]. Importantly, in CHECK-HF we not only collect data on presence or absence of medication, but also on the exact prescribed dosage. Knowledge regarding adherence to current recommended treatment, including dosage, is important as it can provide new insights on how we can improve current prescription rates and current practice.

The current study population of chronic HF patients in a real word setting differs from clinical trials by the high percentage of female patients and elderly patients and high prevalence of multiple co-morbidities. These categories are chronically underrepresented in clinical trials, but form a significant part of the HF population seen in daily practice. The CHECK-HF registry will evaluate these subgroups with specific attention in secondary analyses studying treatment differences or differences in co-morbidity distribution.

### The CHECK-HF registry

The CHECK-HF registry consisted of a relatively high proportion of HFpEF patients (21%). As noted in previous literature, there is a high percentage of female and/or elderly patients in this subgroup. The ESC guidelines had no specific treatment recommendations for HFpEF due to lack of evidence in randomised clinical trials. The CHECK-HF registry will describe how these patients are treated and evaluate the distribution of age, gender and co-morbidity within this subgroup of HFpEF patients.

The number of co-morbid conditions in a patient with chronic HF is high and has been increasing in the last decade [11]. The majority of patients have 5 or more co-morbid chronic conditions which are important determinants of the number of hospital re-admissions [11]. We can argue that in order to reduce the number of HF re-admissions, we should focus on influencing the level of co-morbidities [11]. We can also hypothesise that earlier interventions could lower the extent of co-morbidities and collateral damage. There is a variety of examples of relatively simple solutions which may affect prognosis such as iron supplementation in iron deficient HF patients. Other examples are vitamin D deficiency, obstructive sleep apnoea syndrome and subclinical hypothyroidism. Secondary analyses of the CHECK-HF registry will focus on the level of co-morbidities in a real-world cohort and study potential interaction with the level of co-morbidities and treatment adherence. However, given the set-up of the CHECK-HF registry, we only have information for a selected number of co-morbidities.

### Comparison with other registries

Several large national HF registries have been published in Europe [6–9, 12–17]. In Tab. 2 we provide an example of relevant major European HF registries with more than thousand patients with a report on heart failure medication and/or ICD therapy in specific patients with chronic heart failure [6–9, 12–17]. We particularly want to address two registries. The European Society of Cardiology Heart Failure (ESC-HF) pilot study was a prospective, multicentre, observational survey conducted in 136 cardiology centres from 12 European countries selected to represent the different health systems and care attitudes across Europe [7]. The ESC-HF pilot 2010 included 3,226 patients with chronic HF; mean age was 67 years and percentage of female patients was 29.7%, both figures much lower than in the CHECK-HF registry and significantly lower than expected in a real-world HF population [7]. The adherence to recommended therapy in HF was 88% for ACE inhibitors or ARBs, 87% for beta-blockers, and 44% for MRA, but no distinction was made between patients with reduced versus preserved LVEF regarding therapy [7]. Moreover, there is limited available information on dosage, which will be an important strength of CHECK-HF. The ESC-HF long-term registry (ESC-HF-LT) was a prospective, observational registry in 211 cardiology centres from 21 European and/or Mediterranean countries, all being member countries of the ESC [8]. Between May 2011 and April 2013, it collected data on 12,440 patients, 40.5% of them hospitalised with acute HF and 59.5% outpatients with chronic HF. Thus, the ESC-HF-LT registry evaluated 7,401 patients with chronic HF with a mean age of 66 years and 28.8% female patients. Again, these figures are much lower compared with the current real world HF registry in CHECK-HF [8, 9]. The mean EF was 35%. Overall adherence to HF medication was high with 89% for ACE inhibitors, 88% for beta-blockers, and 59% for MRA, but again no information on appropriateness of their use. The ARNO Observatory study by the Italian National Health Service (INHS) consisted of 41,413 patients who were discharged for HF from 1 January 2008–31 December 2012 and prescribed at least one HF treatment [18]. ACE inhibitors/ARBs, beta-blockers, and mineralocorticoid antagonists were prescribed in 65.8%, 49.7%, and 42.1% of patients respectively. During 1‑year follow-up, at least one rehospitalisation occurred in 56.6% of patients, 49% of them due to non-cardiovascular causes [18].

**Table 2:** Overview of relevant large scale registries forchronic heart failure care programmes in European countries

Registry of chronic HF patients	Cohort size^*^	Inclusion period	Publication date	Mean age	Gender, female	Ischaemic aetiology	Comorbidity, HT vs DM %	Medication ACE/ARB; BB; MRA	ICD/CRT-D therapy, %
EuroHeartFailure survey [6]	11,304	2001–2003	2003	71	47	39	n/a vs 20%	62%; 37%; 21%	n/a
ESC-HF Pilot study (*n* = 5118) [7]	3226	2009–2010	2010	67	30	50	58% vs 29%	88%; 87%; 44%	ICD 13%, CRT-D 9%
ESC-HF-Long-term-Registry [8, 9]	7401	2011–2013	2013	66	29	43	58% vs 32%	89%; 89%; 59%	n/a
The Swedish Heart Failure Registry (S-HFR), subset with EF < 30% [12]	5908	2003–2012	2016	72	23	61	34–48% vs 70%	88%; 85%; 53%	4–10%
Evidence-based Treatment in HF registry (Germany): EVITA-HF [13]	1853	2004–2011	2014	70	24	56	76% vs 39%	73%; 71%; 32%	18%
Spanish National Registry on Heart Failure: the RICA registry [14]	1368	2008–2013	2015	78	53	n.a.	86% vs 47%	87%; 64%; 32%	n/a
Italian Network on Heart Failure Outcome registry IN-HF (*n* = 5610) [15]	3755	2007–2009	2013	69	24	46	43% vs 30%	Not specified	ICD 19%, CRT-D 7%
Adherence to guidelines in chronic HF: the MAHLER survey [16]	1410	2001–2002	2005	69	31	68	n/a	69%; 53%; 28%	n/a
Data from the GWTG-HF (Get With The Guidelines) registry [17]	10,148	2005–2007	2009	68	36	15*	n/a	n/a	20%
The QUALIFY international registry [19]	6669	2013–2014	2017	63	26	n/a	64% vs 34%	87%; 87%; 69%	ICD 12.1%, CRT-D 7.2%

*cohort size related to chronic heart failure with reported medication use

*ACE* enzyme inhibitor,* ARB* angiotensin II receptor blocker,* BB* beta-blocker, *DM* diabetes mellitus*, HF* heart failure, *HT* hypertension, *ICD* implantable cardioverter defibrillator, *CRT-D* cardiac resynchronisation therapy defibrillator, *MRA* mineralocorticoid receptor antagonist, *n/a* not available

## Strengths of the CHECK-HF registry

Overall, the CHECK-HF registry has several strengths. The CHECK-HF registry is one of the largest of its kind with almost 11,000 patients included. Moreover, it reflects actual real-world care as performed on outpatient HF clinics with a larger proportion of females and elderly when compared with other registries and clinical trials in particular, which better represents actual daily practice in many hospitals. The CHECK-HF registry has detailed information on medication use and dosage. The CHECK-HF registry also has information on co-morbidity levels and some biomarkers. Because there is extensive detailed information available, the CHECK-HF registry can provide more insight in relatively large subgroups of patients with HFpEF or HFmrEF and specific subsets of patients with atrial fibrillation and heart failure. A limitation of our study is the lack of detailed follow-up data. We plan to collect longitudinal data in the near future to report on the quality of HF care in the Netherlands and intend to perform several cross-sectional follow-ups of outpatient clinics in the Netherlands, with the perspective of repeated analyses of CHECK-HF as a long-term HF care research project. Longitudinal post-hoc data can potentially be obtained from mortality data from Dutch national archives (Statistics NL – CBS).

In conclusion, the CHECK-HF registry is a large HF registry which enrolled nearly 11,000 unselected patients with chronic HF treated at an outpatient clinic setting in the Netherlands to evaluate current HF management in 2013–2016 in a real-world setting. Specific attention will be given to gender and age differences and level of co-morbidities regarding HF treatment and guideline adherence in future analyses.
